# From Morphologic Obscurity to Diagnostic Clarity in Bone Marrow Necrosis: A Clinicopathological Approach to Diagnosis and Etiological Assessment

**DOI:** 10.22034/ijp.2026.2087239.3644

**Published:** 2026-08-01

**Authors:** Seetu Palo

**Affiliations:** 1 Department of Pathology and Laboratory Medicine, All India Institute of Medical Sciences, Bibinagar, Telangana, India

## Dear Editor,

Bone marrow necrosis (BMN) is an uncommon but clinically significant pathological entity characterized by extensive destruction of hematopoietic tissue and the marrow stromal framework while typically preserving cortical bone(1). The reported incidence ranges from approximately 0.3% to 2% in bone marrow biopsies, although the true prevalence may be underestimated due to sampling limitations(1). The pathogenesis of BMN is multifactorial and largely related to ischemic injury to the marrow microenvironment (Table 1).

**Table 1:** Major Causes of Bone Marrow Necrosis

Etiologic Category	Specific Conditions / Examples	Pathogenetic Mechanism
Hematologic malignancies	Acute lymphoblastic leukemia (ALL), acute myeloid leukemia (AML), high-grade non-Hodgkin lymphoma, chronic myeloid leukemia in blast crisis	Marrow infiltration, microvascular compression, cytokine-mediated endothelial injury
Metastatic solid tumors	Carcinomas of prostate, breast, lung, stomach, pancreas; melanoma	Tumor infiltration causing vascular compromise and ischemia
Hemoglobinopathies and hematologic disorders	Sickle cell disease, severe hemolytic anemia, myeloproliferative neoplasms	Vaso-occlusion and ischemic injury to marrow microcirculation
Infections	Bacterial sepsis, tuberculosis, fungal infections (e.g., Aspergillus, Candida), viral infections	Inflammatory cytokine release, septic microvascular thrombosis
Drugs and therapy-related causes	Chemotherapy, interferon therapy, granulocyte colony-stimulating factor (G-CSF), certain antibiotics	Direct cytotoxicity or vascular injury
Autoimmune and systemic conditions	Antiphospholipid syndrome, systemic lupus erythematosus, disseminated intravascular coagulation	Microthrombi and vascular occlusion
Metabolic and toxic causes	Severe hypoxia, alcoholism, severe malnutrition	Impaired oxygen delivery and metabolic stress
Idiopathic cases	No identifiable cause	Likely multifactorial or occult disease

Disruption of the marrow vascular supply leads to hypoxia and necrosis of hematopoietic cells and stromal components(2). One of the principal mechanisms involves vascular compromise caused by infiltration of the marrow by malignant cells. Rapid proliferation of neoplastic cells may compress the sinusoidal network, impairing blood flow and causing local ischemia. In addition, malignant cells can produce cytokines and pro-inflammatory mediators that induce endothelial damage and microvascular thrombosis. Cytokine-mediated injury also contributes to endothelial dysfunction and disturbances in marrow microcirculation. Hematologic malignancies represent the most common etiologic group associated with BMN, particularly acute leukemias and high-grade lymphomas(2-4). Metastatic solid tumors, most frequently from prostate, breast, stomach, and lung, may also lead to extensive marrow necrosis. Non-neoplastic causes include sickle cell disease, in which vaso-occlusive crises produce marrow ischemia and necrosis(1,2,5). Severe infections such as bacterial sepsis, tuberculosis, and fungal infections can result in inflammatory vascular injury and subsequent marrow necrosis(5,6). Drug-related cases have also been reported, particularly in association with chemotherapeutic agents and granulocyte colony-stimulating factor(7).

Recognition of BMN is important because it often reflects severe systemic disease and may be associated with poor clinical outcomes. In some cases, it represents the first pathological clue prompting investigation for an underlying malignancy or systemic disorder. Diagnostic evaluation can be challenging because necrosis may obscure the underlying pathology and marrow aspiration frequently yields insufficient material. Clinically, BMN presents with nonspecific manifestations related to marrow failure and the underlying disease process. Severe bone pain, especially involving the axial skeleton, is the most common presenting symptom. Patients may also present with fever, malaise, and constitutional symptoms(8). Peripheral blood findings often demonstrate cytopenias affecting one or more cell lineages, with anemia and thrombocytopenia being particularly frequent(2,8). A leukoerythroblastic blood picture may be observed, characterized by circulating nucleated red blood cells and immature myeloid precursors. Laboratory studies commonly reveal markedly elevated serum lactate dehydrogenase levels reflecting tissue necrosis(5). Bone marrow aspiration frequently results in a “dry tap” due to replacement of normal marrow with necrotic material, and aspirated material, when obtained, may consist largely of amorphous debris with poorly preserved cellular elements.

**Fig. 1:**
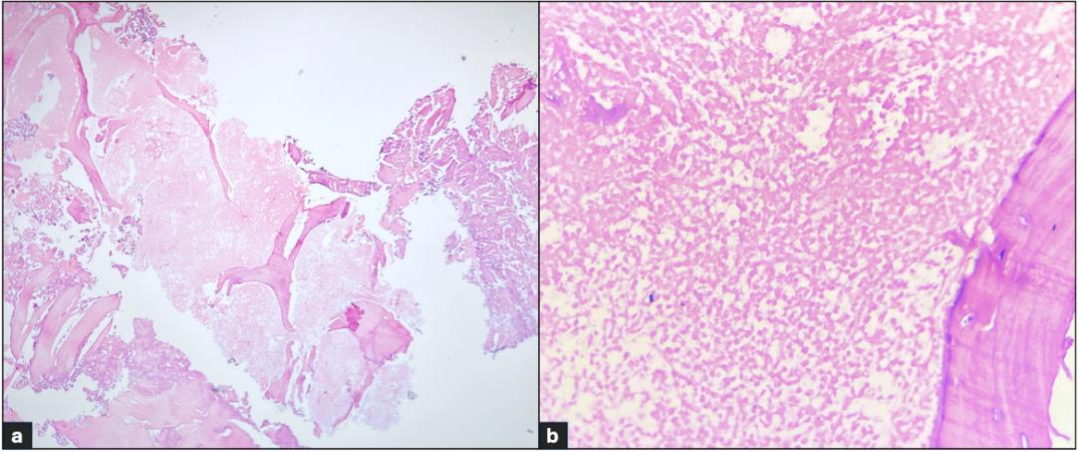
(a) Low-power view of the bone marrow biopsy showing extensive areas necrosis with loss of normal hematopoietic elements and preservation of bony trabeculae (H&E, x40); (b) Higher-power view demonstrating ghost outlines of necrotic hematopoietic cells (H&E, x400).

Histopathological examination of a bone marrow biopsy remains the cornerstone for diagnosis. The defining feature is loss of normal marrow architecture with replacement by eosinophilic, granular, or amorphous necrotic debris(5). At low magnification, the marrow appears extensively disrupted with poorly defined cellular outlines and absence of recognizable hematopoietic elements, while trabecular bone architecture is usually preserved (Fig. 1). At higher magnification, ghost outlines of hematopoietic cells and stromal elements can be identified within an eosinophilic background. Inflammatory infiltrates may be present at the periphery of necrotic zones, and occasional residual viable marrow islands may reveal the underlying disease process such as leukemia or metastatic carcinoma. Several grading systems have been proposed based on the extent of necrosis. A commonly used classification categorizes BMN into Grade I (necrosis involving <20% of marrow), Grade II (20–50%), and Grade III (>50%)(2). This grading may reflect disease severity and has potential prognostic implications.

**Fig. 2:**
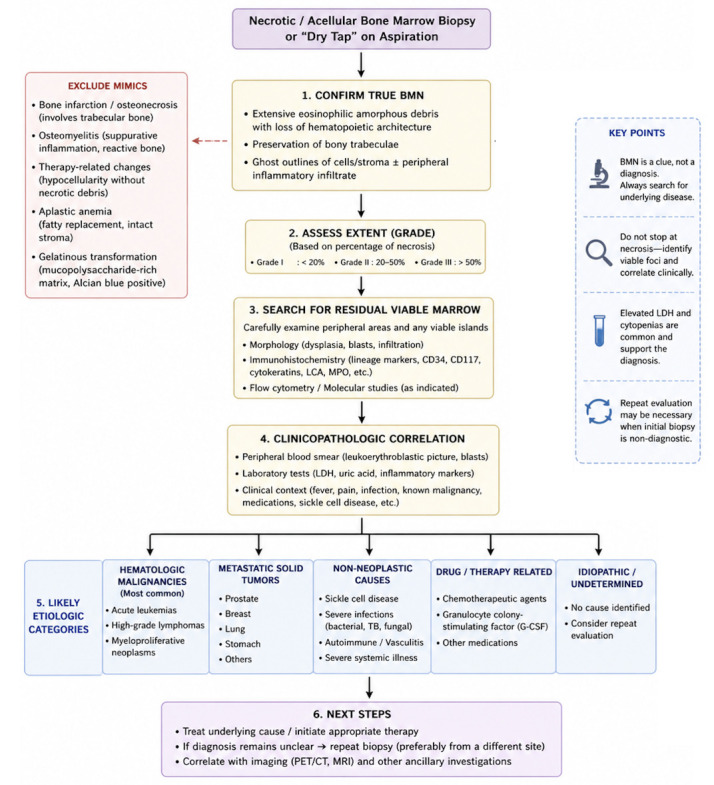
Diagnostic approach to bone marrow necrosis.

The differential diagnosis or the diagnostic pitfalls of BMN includes several conditions that may mimic its histologic appearance. Bone infarction or osteonecrosis represents an important consideration, however, infarction typically involves both marrow and trabecular bone, whereas BMN predominantly affects hematopoietic tissue with preservation of bony architecture. Osteomyelitis may produce marrow destruction but is usually accompanied by suppurative inflammation and reactive bone formation. Furthermore, osteonecrosis and osteomyelitis are typically diagnosed using imaging studies, and biopsy is seldom required for confirmation. Therapy-related marrow changes following chemotherapy or radiation may produce hypocellularity and stromal alterations but lack the characteristic amorphous necrotic background seen in BMN. BMN should also be distinguished from conditions such as aplastic anemia and gelatinous transformation, which may also show loss of hematopoietic elements or homogeneous proteinaceous material(5). In aplastic anemia, the marrow is markedly hypocellular with replacement by fat and stromal elements, while stromal cells remain intact and necrotic debris or cellular shadows are absent. In contrast, gelatinous transformation is characterized by fat cell atrophy, reduced hematopoietic cells, and extracellular deposition of mucopolysaccharide-rich gelatinous material containing hyaluronic acid, which is Alcian blue (pH 2.5) positive and periodic acid Schiff positive, unlike BMN, which is negative. Fig. 2 outlines a stepwise approach to the evaluation of BMN.

In conclusion, BMN is an uncommon but important pathological entity that often signifies severe systemic disease. Accurate diagnosis of BMN requires integration of clinical findings, laboratory parameters, imaging, and histopathological assessment. Careful evaluation of any residual viable marrow is essential to identify diagnostic clues, as extensive necrosis may obscure the underlying pathology. Pathologists should maintain a high index of suspicion for malignancy—particularly acute leukemias and metastatic carcinoma, which are among the most common causes of BMN. Recognition of characteristic features, such as amorphous eosinophilic necrotic debris and ghost outlines of hematopoietic cells, and appropriate reporting can provide an important clue to serious systemic disease. When the initial biopsy is non-diagnostic, close clinicopathologic correlation and, if necessary, repeat sampling are crucial to establish the underlying cause and guide management.

## Data Availability

Data sharing is not applicable to this article.
